# Interleukin-27 Regulates the Function of the Gastrointestinal Epithelial Barrier in a Human Tissue-Derived Organoid Model

**DOI:** 10.3390/biology11030427

**Published:** 2022-03-11

**Authors:** Daniel P. Brice, Graeme I. Murray, Heather M. Wilson, Ross J. Porter, Susan Berry, Scott K. Durum, Mairi H. McLean

**Affiliations:** 1 School of Medicine, Medical Sciences and Nutrition, University of Aberdeen, Aberdeen AB25 2ZD, UK; daniel.brice@manchester.ac.uk (D.P.B.); graeme.murray1@abdn.ac.uk (G.I.M.); h.m.wilson@abdn.ac.uk (H.M.W.); s.h.berry@abdn.ac.uk (S.B.); 2 Centre for Inflammation Research, Queens Medical Research Institute, University of Edinburgh, Edinburgh EH16 4TJ, UK; 3 Cytokines and Immunity Section, Laboratory of Cancer Immunometabolism, National Cancer Institute (NCI), National Institute of Health (NIH), Frederick, MD 21702, USA; durums@mail.nih.gov; 4 Division of Molecular & Clinical Medicine, School of Medicine, University of Dundee, Dundee DD1 9SY, UK

**Keywords:** IBD, colitis, cytokine, interleukin-27, epithelium, organoid, gastrointestinal, permeability

## Abstract

**Simple Summary:**

The gut is lined by a single layer of epithelial cells, creating a barrier between the contents of the gut and the underlying tissue. This barrier also controls permeability (to determine if gut contents move to the tissue) and produces proteins that help with immune responses. These epithelial cells are damaged and do not function properly in inflammatory bowel diseases (IBD). IBD is common, and current treatments are not always effective and can have side effects. New treatments are needed. An alternative treatment target is the epithelial gut lining to promote healing. We used an experimental model of human gut tissue epithelial cells (called organoids) and aimed to define the role of the protein interleukin-27 (IL-27) in epithelial barrier function. We show that IL-27 restored permeability associated with changes in several proteins involved in this process, led to changes in genes involved in the immune response and reaction to bugs in the gut and led to enhanced healing and repair of an injury wound in the barrier. In summary, this study demonstrates a new function of IL-27 in the gut to aid epithelial barrier repair. This is a potential new treatment strategy for IBD.

**Abstract:**

A treatment with direct healing effects on the gastrointestinal epithelial barrier is desirable for inflammatory bowel disease (IBD). Interleukin-27 (IL-27) is an immunoregulatory cytokine, and oral delivery is an effective treatment in murine models of IBD. We aimed to define IL-27 effects on the human gastrointestinal epithelial barrier. We characterised gene and protein expression of permeability mediators in a human colon-derived organoid model. Functional permeability was determined in an organoid-derived 2D monolayer by transepithelial electrical resistance. IL-27 effects on epithelial innate immune responses were assessed through expression of cytokines, anti-microbial peptides and MUC genes. IL-27 effects on wound healing and proliferation were determined in human colon epithelial cell lines. IL-27 led to restoration of permeability regulation following inflammatory cytokine insult (*p* = 0.001), associated with differential expression of tight junction mediators with decrease in claudin 2 (*p* = 0.024) and increase in claudin 4 (*p* < 0.001), E-cadherin (*p* < 0.001) and zona occludens (*p* = 0.0014). IL-27 evoked differential gene expression of epithelial-derived innate immune responses (reduced IL1B and IL18, and increased IL33, HBD1, MUC1 and MUC2; *p* < 0.012). IL-27 induced epithelial barrier wound healing through restitution (*p* < 0.001), and increased proliferation (*p* < 0.001) following injury. Overall, IL-27 provokes mucosal healing of the human gastrointestinal epithelial barrier.

## 1. Introduction

The aim of our study was to define the effect of the cytokine interleukin-27 (IL-27) on the human gastrointestinal epithelial barrier. The gastrointestinal epithelial barrier is a main interface between environment and host, with tightly regulated permeability and rapid restitution of wounds. This barrier comprises several cell phenotypes including the most abundant intestinal epithelial cell, alongside specialised cell types such as goblet cells and Paneth cells, producing mucus for the glycocalyx and anti-microbial peptides, respectively. Cells arise from the Lgr5+ stem cells located at the base of the crypt, differentiating as they migrate towards the luminal surface, where they are shed. This is tightly regulated by a milieu of autocrine and paracrine growth factors derived from supporting mesenchymal cells or the epithelial cells themselves, supporting a rapid turnover of the barrier in entirety approximately every 5 days [1,2]. The epithelial barrier secretes epithelial cell-derived cytokines, anti-microbial peptides and the MUC proteins that create the scaffold for the overlying mucus layer. There is cross-talk between host immune cells, luminal microbiota and the epithelial barrier, and integrity of structure and function of the barrier is key for gastrointestinal homeostasis [2,3]. Disruption of this barrier is associated with the pathogenesis of several pathologies, including inflammatory bowel disease (IBD) [2,4]. 

The incidence of IBD is increasing worldwide [5] and conveys significant morbidity. The pathogenesis includes genetic susceptibility, disruption of the epithelial barrier, microbial dysbiosis and a chronic mucosal immune response leading to relapsing-remitting inflammation of the gastrointestinal tract. There is no cure and current treatments aim to dampen the mucosal inflammatory responses. Despite several available treatment strategies including biologic therapy, these are not effective for all, can lose efficacy over time or cannot be tolerated due to systemic side effects. Therefore, there is a clinical need for new treatment strategies. Previously, we demonstrated that orally administered interleukin-27 (IL-27), luminally delivered to the gastrointestinal mucosa via a *Lactococcus lactis* bacterial vector, was an effective treatment to attenuate IBD across several mouse models of the disease, including a T-cell transfer model of enterocolitis, acute and chronic DSS-induced colitis and acute severe TNBS-induced colitis [6,7]. 

IL-27 is an immunoregulatory cytokine with the capacity to promote CD4+ T-cell proliferation and Th1 polarisation but is also able to suppress immune responses through inhibition of Th2 and Th17 T-cell responses. IL-27 can also induce Tr1 regulatory T cells with subsequent amplification of anti-inflammatory cytokine IL-10 expression [8]. IL-27 can also promote intra-epithelial T cell responses in the gut [9]. In the mouse models, treatment effect was induced through multiple mechanisms including induction of IL-10 expression from gastrointestinal T cells, reduction in pro-inflammatory cytokine responses and reduced chemokine gradient with reduction in neutrophil migration [6,7]. Mice treated with IL-27 had less mucosal ulceration histologically, with immunohistochemical evidence of phosphorylation of STAT3 in gastrointestinal epithelial cells [7], suggesting that IL-27 had a direct impact on the gastrointestinal epithelial barrier. The biological consequences of this were not explored and are unknown. 

Given that defective barrier structure and function are present in active IBD and that clinical treatment aims to achieve endoscopic mucosal healing, a new treatment that is not only immunoregulatory but also promotes epithelial barrier healing directly is desirable. Therefore, leading on from the pre-clinical data, our aim was to define the effect of IL-27 on the human gastrointestinal epithelial barrier. This has not been investigated previously. We used human tissue-derived organoid modelling to achieve this. We show that IL-27 promotes wound healing of the epithelial barrier through restitution following injury. IL-27 restores permeability regulation following an inflammatory cytokine insult, associated with differential gene and protein expression of key mediators of permeability at rest and under inflammatory conditions. Moreover, IL-27 evokes differential gene expression of mediators to bolster innate immune responses such as cytokines, anti-microbial peptides and MUC proteins for the mucus layer. 

## 2. Materials and Methods

### 2.1. Ethical Approval and Tissue Source

Ethical approval for use of human tissue was obtained from the Grampian Tissue Biorepository (Tissue Request No. 000065 via delegated authority (11/NS/0015) from The North of Scotland research ethics committee to approve research involving human tissue and data). All tissue was anonymised. Patients provided written consent. Tissue samples were obtained from patients undergoing colorectal surgery for treatment of colorectal cancer. Full thickness segments of non-neoplastic colonic mucosa from the resection margins were prepared by an expert gastrointestinal pathologist (GIM).

### 2.2. Colorectal Cell Line Culture

Caco-2 (ATCC^®^ HTB-37™) and HT-29 (ATCC^®^ HTB-38™) cell lines were obtained from ATCC, LGC Standards, Middlesex, UK and authenticated by short tandem repeat (STR) profiling (Eurofins Genomics, Ebersberg, Germany). Cells were cultured in high-glucose (4500 mg/L) Dulbecco’s modified Eagle’s media (DMEM) Gibco^®^ Invitrogen™ Life Technologies Ltd., Paisley, UK, containing 10% foetal bovine serum (FBS), 4 mM L-glutamine and a mixture of 50 IU/mL penicillin and 50 μg/mL streptomycin. In addition, Caco-2 cells were supplemented with 0.1 mM MEM NEAA (non-essential amino acids). Cell lines were incubated at 37 °C, with 5% carbon dioxide (CO_2_) and constant humidity. 

### 2.3. Human Colon-Derived Epithelial Organoid Model

#### 2.3.1. 3D Organoid Culture

A human colon-derived epithelial organoid model was established as we previously described [10] from a published protocol [11]. Human colonic mucosa was chelated in 2.5 mM EDTA buffer on ice for 40 min with gentle agitation, then passed through a 100 μM cell strainer. Following centrifugation and washing, crypts were suspended in Matrigel© Corning^®^, New York, NY, USA, supplemented with 1:100 Jagged-1-peptide at 200 crypts/15 μL Matrigel© Corning^®^, New York, NY, USA. A total of 15 μL crypt-Matrigel© Corning^®^, New York, NY, USA mixture per well was dispensed into a pre-warmed delta-surface 24-well plate, and the Matrigel© Corning^®^, New York, NY, USA was polymerised by inverting plate at 37 °C for 15 min. For initiating, maintaining and differentiation of colonic organoid culture, a 50% conditioned media (1:1 dilution in advanced D-MEM/F12 base media) was generated from genetically modified L-WRN mouse fibroblast cell line (ATCC^®^, CLR-3276^™,^ ATCC, LGC Standards, Middlesex, UK) cultured in advanced D-MEM/F12 with 10% FCS, 0.5 mg/mL hygromycin-B and 0.5 mg/mL G-418. Initiation media contained 1% bovine serum albumin, 2 mM Glutamax, 2 mM L-glutamine, 10 mM HEPES, 1× N2, 1× B27, 1 mM N-acetylcysteine, 50 ng/mL rhEGF, 10 mM nicotinamide, 500 nM A83-01, 10 nM prostaglandin E2, 10 nM [Leu-15]-gastrin1, 10 μM SB202190, 2.5 μM Thiazovinin, 10 μM Y-27632 dihydrochloride, 2.5 μM CHIR99021 and 100 μg/mL Primocin. Maintenance media was prepared with the same constituents minus 10 μM Y-27632 dihydrochloride and 2.5 μM CHIR99021. Differentiation media was prepared as per initiation media minus 10 mM nicotinamide, 10 μM SB202190, 10 μM Y-27632 dihydrochloride and 2.5 μM CHIR99021. Prior to experiments, organoids were cultured in 100% DMEM/F12 with the same additives as per differentiation media, minus 10 nM prostaglandin E2 and 2.5 μM Thiazovinin and with added human recombinant Wnt3A 50 ng/mL, R-Spondin-1250 ng/mL and noggin 50 ng/mL. Organoids were cultured at 37 °C in 5% CO_2_ in initiation media for 48 h, then replaced with maintenance media, refreshed every 3–4 days. Before experimental stimulations, organoids were plated on delta-surface 96-well plates and cultured in fully recombinant differentiation media for at least 3–4 days to maturity.

#### 2.3.2. Human Colon Organoid-Derived Monolayer Culture

Human colon-derived organoid culture was used to create a 2D monolayer using a modified protocol from a published porcine tissue-derived model [12]. Organoid containing Matrigel^®^ domes were scratched from the well and pooled, and then the organoids were released by shaking at 25 rpm in 15 mM EDTA in DPBS for 15 min at 4 °C before centrifuging at 300× *g* for 3 min at 4 °C. The pellet was resuspended in 1 mL Trypsin-EDTA (0.25%), incubated for 10 min, then neutralised with four volumes of DMEM/F12 + 10% FBS and centrifuged at 1000× *g* for 5 min. The pellet was resuspended in 1 mL DMEM/F12 10% FBS, and 4 mL DMEM/F12 10% FBS was added and then the mixture was centrifuged at 1000× *g* for 5 min. The pellet was resuspended in initiation media to a final density of 1.5 × 10^5^ cells/mL. For 2D monolayer preparation, wells in a Corning™ (USA) Transwell PET 0.4 µm 12 mm culture plate were coated with 1% (*v*/*v*) Matrigel^®^ in DMEM/F12 to create a basement matrix for adherence and were incubated at 37 °C for 1 h. To each well, 500 μL of organoid-derived cell suspension was added to the apical chamber and 800 μL initiation media was placed in the basolateral chamber, then they were cultured at 37 °C 5% CO_2_ and constant humidity. 

### 2.4. Organoid Stimulation Assays and Ex Vivo Inflammatory Organoid Model

Gene and protein expression in organoids were assessed following 24 or 48 h stimulation, respectively, with 100 ng/mL recombinant human IL-27 (rhIL-27) alone or under inflammatory conditions created by the addition of rTNF (100 ng/mL) and LPS (500 ng/mL). 

### 2.5. Gene Expression Analysis 

RNA was extracted from organoids (*n* = 3–5 individual patients with 3 technical replicates/experiment) using an RNeasy^®^ Plus Mini Kit (Qiagen, Crawley, UK) with cDNA prepared using a SuperScript^™^ VILO^™^ cDNA synthesis kit (Invitrogen, Paisley, UK). Expression of TLR4 (forward: 5′-CAGAGTTGCTTTCAATGGCATC-3′, reverse: 5′-AGACTGTAATCAAGAACCTGGAGG-3′; 282 bp, NM_003266.4) and IL-27Rα (forward: 5′-GCCTTCTGCTCCAAAAGATG-3′, reverse: 5′-GGAGCAGCAGCAGGTAATTC-3′; 175 bp, NM_004843.4) were assessed by endpoint PCR incorporating SYBR^®^ Green technology and the product visualised by ethidium bromide incorporation on a 1% agarose/Tris borate EDTA gel. RT-qPCR using TaqMan assays (Applied Biosystems, Thermo Fisher Scientific UK Ltd., Loughborough, UK) characterised colonic organoid permeability genes (CLDN2, CLDN4, OCLN, TJP1, CDH1), epithelial-derived cytokine (IL1β, IL18, IL33, IL10, IL22, IL27), anti-microbial peptide (HBD1) and MUC 1 and 2 gene expression normalised to GAPDH and B2M on a StepOnePlus™ real-time PCR system (Applied Biosystems, Thermo Fisher Scientific UK Ltd., Loughborough, UK). Relative gene expression was calculated using the Livak Method [13].

### 2.6. Protein Extraction and Immunoblotting

Organoids were incubated in 1X RIPA buffer plus 10 μL Halt^™^ protease inhibitors on ice for 30 min, then agitated using a needle and syringe. Protein concentration was determined using a Pierce^™^ BCA Protein Assay Kit (Thermo Fisher Scientific UK Ltd., Loughborough, UK). Expression of proteins was performed by Western blotting using a semi-automated Amersham^™^ WB system (GE Healthcare UK Ltd., Little Chalfont, UK) incorporating a 8–18% SDS-PAGE gel card, normalised to endogenous GAPDH (mouse IgG1, 1:5000, Abcam (ab8245)) protein expression. Proteins assessed were claudin-2 (rabbit IgG, 1:500, ab53032), claudin-4 (rabbit IgG, 1:1000, ab210796), E-cadherin (rabbit IgG, 1:10,000, ab40772) and occludin (rabbit IgG, 1:1000, ab216327), all purchased from Abcam. Secondary antibodies were purchased from GE Healthcare (mouse IgG, Cy^™^3, 1:2500, and rabbit IgG, Cy^™^5, 1:2500). Data were analysed using the Amersham^™^ WB Software (GE Healthcare UK Ltd., Little Chalfont, UK) with endogenous protein normalisation of samples and normalisation to negative controls. 

### 2.7. Epithelial Barrier Permeability Assay 

Functional permeability across a human colon organoid-derived monolayer was established by transepithelial electrical resistance (TEER) using a EVOM2 transepithelial electrical resistance meter (World Precision Instruments, Hitchin, UK). When cells reached confluence, TEER measurements were recorded every 24 h until they were stabilised for 2 days at 1200–1300 Ohms/cm^2^; then, stimulations were performed in triplicate. Three TEER measurements were taken for each sample at each timepoint.

### 2.8. Wound Healing Assay

Wound healing was assessed by a scratch assay as previously published [14,15]. Reference lines were marked on a 6-well cell culture plate. Caco2 and HT-29 cells were seeded at a density of 1.5 × 10^6^ cells per well and cultured to confluence. A sterile p200 pipette tip was used to create a linear ‘wound’, perpendicular to the pre-marked reference line, *n* = 3 with 3 technical replicates per experiment, and cells were stimulated with 50 or 100 ng/mL rhIL-27. Photomicrographs were taken from each side of the reference line (EVOS Microscope, Thermo Fisher Scientific UK Ltd., Loughborough, UK) at 0, 24, 48 and 72 h. ImageJ Software (Available online: https://imagej.nih.gov (last accessed on 22 December 2021)) was used to calculate wound reconstitution rates on the basis of wound images at time 0. 

### 2.9. Proliferation Assay

Caco2 and HT-29 cells were seeded in a 12-well plate at a density of 1 × 10^5^ cells per well and cultured for 24 h. Proliferation was measured after 24, 48 or 72 h stimulation with 50 or 100 ng/mL rhIL-27 or 50 ng/mL EGF as a positive control, *n* = 3 with 3 technical replicates. A total of 4 µCi tritiated thymidine (GE Healthcare Ltd., Little Chalfont, UK) per well was added 24 h prior to stimulation end time [16]. Cells were harvested onto glass-fibre filter mats using a Mach III Harvester 96 (Tomtech, Spalding, UK). Mats were dried, placed in filter pockets with beta-scintillation fluid (PerkinElmer Inc., Buckinghamshire, UK) and radioactivity determined, as counts per minute, on a MicroBeta Trilux scintillation counter (PerkinElmer Inc., Buckinghamshire, UK). 

### 2.10. Statistical Analysis

Data are expressed as mean ± standard deviation for the specified number of experiments (*n*). Normality tests (Shapiro–Wilk or Kolmogorov–Smirnov) determined whether parametric or non-parametric statistical tests were used. Statistical significance was resolved using Student’s *t*-test or unpaired Mann–Whitney *U* test. Where multiple comparisons were required within an experiment, ANOVA was used with Bonferroni correction. Analyses were performed in Prism v.9.0.2 (GraphPad Prism v.9.0.2, GraphPad Software, La Jolla, CA, USA. Available online: https://www.graphpad.com (last accessed on 22 December 2021)). Statistical significance was denoted as * *p* < 0.05, ** *p* < 0.01, *** *p* < 0.001 and **** *p* < 0.0001.

## 3. Results

### 3.1. IL-27 Regulated Permeability of the Human Gastrointestinal Epithelial Barrier Following Inflammatory Insult

**Figure 1:**
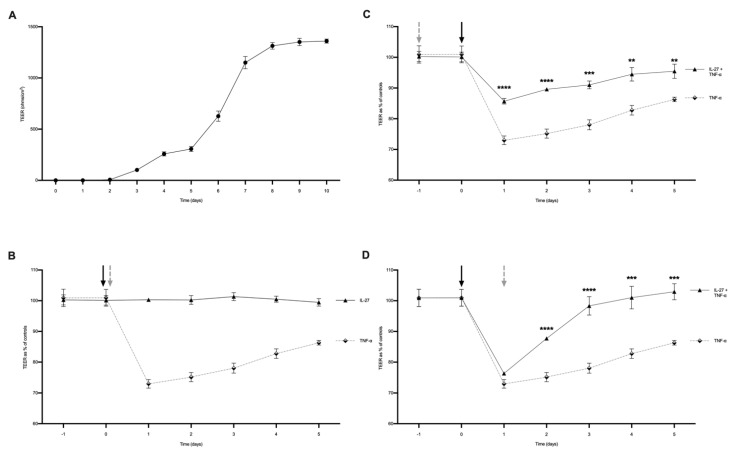
IL-27 effect on paracellular permeability of human organoid-derived monolayers. Paracellular permeability measured by trans-epithelial electrical resistance. (**A**) Human organoid-derived monolayers reached confluence 8 days post-seeding with stable TEERs. (**B**) Stimulation of monolayers with TNF-α (100 ng/mL administered day 0, black arrow) increased paracellular permeability by ≈25% that of unstimulated controls, whereas rhIL-27 (grey arrow) alone did not influence permeability. (**C**) Pre-treatment of monolayers with rhIL-27 (100 ng/mL) 24 h prior (grey arrow) to administration of TNF-α (black arrow) reduced the increase in permeability seen with TNF-α alone. (**D**) Post-treatment of monolayers with rhIL-27 (100 ng/mL) (grey arrow) 24 h after TNF-α administration (arrow) resulted in rapid recovery of paracellular permeability compared to monolayers treated with TNF-α alone. *n* = 3 individual patient-derived organoids with 3 technical replicates/experiment with three TEER measurements were taken for each sample at each timepoint, presented as mean ± SD. Statistical analysis with one-way ANOVA with Bonferroni correction. * *p* < 0.05, ** *p* < 0.01, *** *p* < 0.001 and **** *p* < 0.0001; ns = not significant.

We first assessed whether IL-27 led to a change in functional permeability across the human gastrointestinal epithelial barrier. We developed a human tissue-derived organoid monolayer culture in a Transwell system that allowed for the measurement of transepithelial electrical resistance (TEER) as a marker of barrier integrity and permeability regulation. We confirmed that the human colon organoids expressed the IL-127Rα receptor subunit (Figure S1A), therefore demonstrating that these models had the capacity to respond to IL-27 stimulation. Serial measurements of TEER over time revealed establishment of barrier integrity by day 8 (Figure 1A). This barrier could be rendered ‘leaky’ with increased permeability provoked by TNF exposure [17,18,19] (Figure 1B). TNF is a main pro-inflammatory cytokine present in the mucosa in active IBD and is known to disturb gastrointestinal epithelial barrier function as part of disease pathogenesis [20]. When the epithelial barrier was exposed to IL-27 24 h prior to TNF insult, the barrier was damaged by TNF stimulation, but less so than TNF alone, and barrier integrity was re-established more rapidly over time (Figure 1C, *p* < 0.01). When the epithelial barrier was exposed to IL-27 24 h after TNF insult, there was a very rapid restitution of barrier integrity (Figure 1D, *p* < 0.001). 

### 3.2. IL-27 Led to Differential Expression of Key Mediators of Human Gastrointestinal Epithelial Barrier Permeability and Barrier Integrity in a Human Colon Organoid Model 

**Figure 2:**
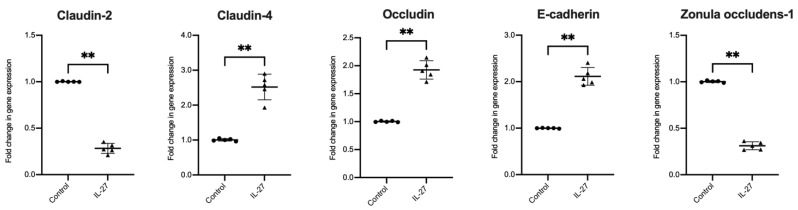
IL-27 effect on human gastrointestinal colonic organoid gene expression. RT-qPCR analysis of human patient-derived organoids stimulated with rhIL-27 (100 ng/mL) compared to unstimulated controls of epithelial barrier function genes. Expression normalised to GAPDH; *n* = 5 individual patient-derived organoids, with 3 technical replicates/experiment, presented as mean ± SD. Statistical analysis with Mann–Whitney *U* test. * *p* < 0.05, ** *p* < 0.01, *** *p* < 0.001 and **** *p* < 0.0001; ns = not significant.

To explore the mechanisms associated with IL-27 regulation of barrier permeability, we next assessed the gene expression of key mediators of epithelial barrier permeability and integrity in the human colon organoid model. Expression of claudin-2 (pore forming, *p* = 0.007) and ZO-1 (tight junction stability, *p* = 0.007) were decreased with IL-27. Claudin-4 (pore sealing, *p* = 0.007), *occludin* (tight junction stability, *p* = 0.007) and E-cadherin (cell-cell adhesion, *p* = 0.007) were significantly increased in response to rhIL-27 (Figure 2). 

**Figure 3:**
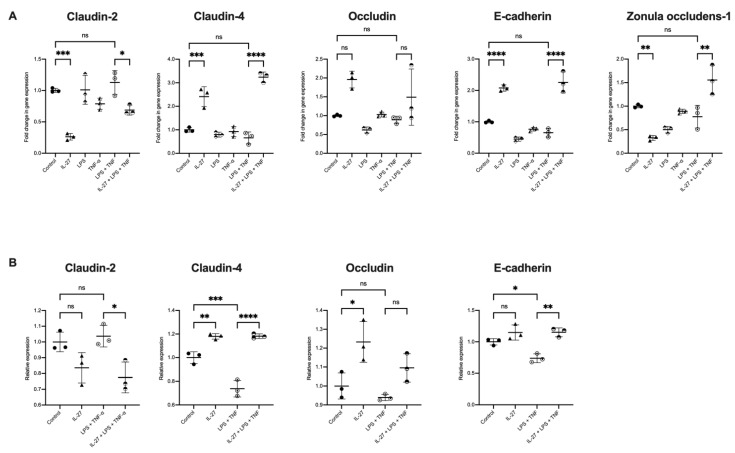
IL-27 effect on human gastrointestinal colonic organoid gene and protein expression in an organoid model of IBD. Human colonic organoids stimulated with rhIL-27 (100 ng/mL), LPS (500 ng/mL), TNF-α (100 ng/mL) and combinations of the three as indicated. (**A**) Expression of epithelial barrier function genes compared to unstimulated controls. (**B**) Protein expression of epithelial barrier junction proteins as determined by Western blotting. Gene and protein expression normalised to GAPDH; *n* = 3 individual patient-derived organoids, with 3 technical replicates/experiment, presented as mean ± SD. Statistical analysis with one-way ANOVA with Bonferroni correction. * *p* < 0.05, ** *p* < 0.01, *** *p* < 0.001 and **** *p* < 0.0001; ns = not significant.

In active IBD, the epithelium is exposed to inflammatory signalling molecules. Epithelial injury and inflammation result in loss of barrier function, exposing the epithelium to microbial antigens that would otherwise be restricted to the lumen under homeostatic conditions. To re-create this microenvironment in an in vitro organoid IBD model, colonic organoids were stimulated concurrently with LPS (500 ng/mL) and/or TNF-α (100 ng/mL). The organoids expressed TLR4, the receptor for LPS (Figure S1B). LPS and TNF stimulation did not impact the expression of the candidate genes compared to untreated controls (Figure 3A). When IL-27 was added to this inflammatory organoid model (LPS + TNF versus LPS + TNF + IL-27), once again there was a differential expression of key genes (Figure 3A). Expression of claudin-2 (pore forming, *p* = 0.0246) was reduced. Claudin-4 (pore sealing, *p* = 0.0001) and E-cadherin (cell-cell adhesion, *p* < 0.0001) were significantly increased in response to IL-27 in the inflammatory environment. There was no change in expression of occludin with IL-27 under inflammatory conditions, whereas expression of ZO-1 (tight junction stability, *p* = 0.0014) was increased. 

The gene expression changes identified in permeability mediators with IL-27 were translated to changes in protein expression assessed by Western immunoblotting (Figure 3B, Table 1). Under inflammatory conditions (with TNF and LPS), protein expression of claudin-2 was decreased (*p* = 0.027), and expression of claudin-4 (*p* = 0.0001) and E-cadherin (*p* = 0.0016) increased with IL-27. 

### 3.3. IL-27 Stimulated Human Gastrointestinal Epithelial Barrier-Derived Innate Immunity

Having established that IL-27 impacts on gastrointestinal epithelial barrier permeability, we explored IL-27 effects on other epithelial barrier functions. The gastrointestinal epithelial barrier contributes to innate immune responses through secretion of cytokines (including IL-1β, IL-18, IL-33, and IL-22), anti-microbial peptides (human β defensin-1 (hBD1) from colon) and the MUC proteins that create the scaffold for the overlying mucus layer. 

**Figure 4:**
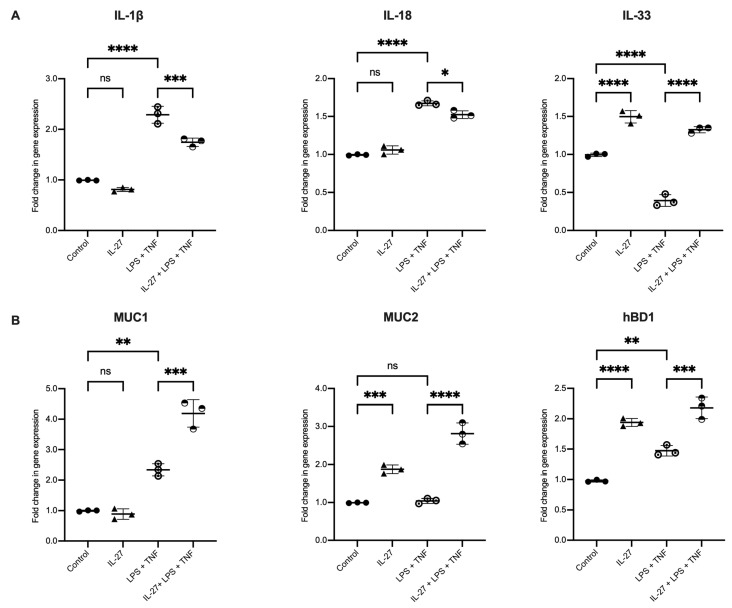
IL-27 stimulated human gastrointestinal epithelial barrier-derived innate immunity. Human colonic organoids stimulated with rhIL-27 (100 ng/mL), LPS (500 ng/mL), TNF-α (100 ng/mL) and combinations of the three as indicated. (**A**) Gene expression of epithelial-derived cytokines compared to unstimulated controls. (**B**) Expression of mucins (muc)−1 and −2 and human beta defensin 1 (hBD1). Gene expression normalised to GAPDH; *n* = 3 individual patient-derived organoids, with 3 technical replicates/experiment, presented as mean ± SD. Statistical analysis with one-way ANOVA with Bonferroni correction. * *p* < 0.05, ** *p* < 0.01, *** *p* < 0.001 and **** *p* < 0.0001; ns = not significant.

IL-27 did not change expression of the cytokines IL-1β or IL-18 (Figure 4A). However, expression of IL-33 was increased (*p* = 0.0001). As expected, the pro-inflammatory cytokines IL-1β (*p* = 0.001) and IL-18 (*p* = 0.001) were increased in response to TNF and LPS. There was a significant reduction in IL-33 expression (*p* = 0.001). Exposure to IL-27 under inflammatory conditions significantly decreased human colonic organoid expression of IL-1β (*p* = 0.0007) and IL-18 (*p* = 0.0127), and increased IL-33 (*p* = 0.0001). The human colon-derived organoids did not express IL-10, IL-22 or IL-27 at rest or in response to inflammatory stimulation.

IL-27 alone did not change expression of MUC1 (Figure 4B). IL-27 significantly increased expression of MUC2 (*p* = 0.0007) and hBD1 (*p* = 0.0001). This pattern was also seen under inflammatory conditions compared to unstimulated organoids, *p* = 0.00023 and 0.0014 for increased hBD1 and MUC1, respectively. IL-27 in inflammatory conditions led to a heightened and significantly increased expression of MUC1 (*p* = 0.0001), MUC2 (*p* = 0.0001) and hBD1 (*p* = 0.0002). 

### 3.4. IL-27 Stimulated Gastrointestinal Epithelial Barrier Wound Restitution and Proliferation

**Figure 5:**
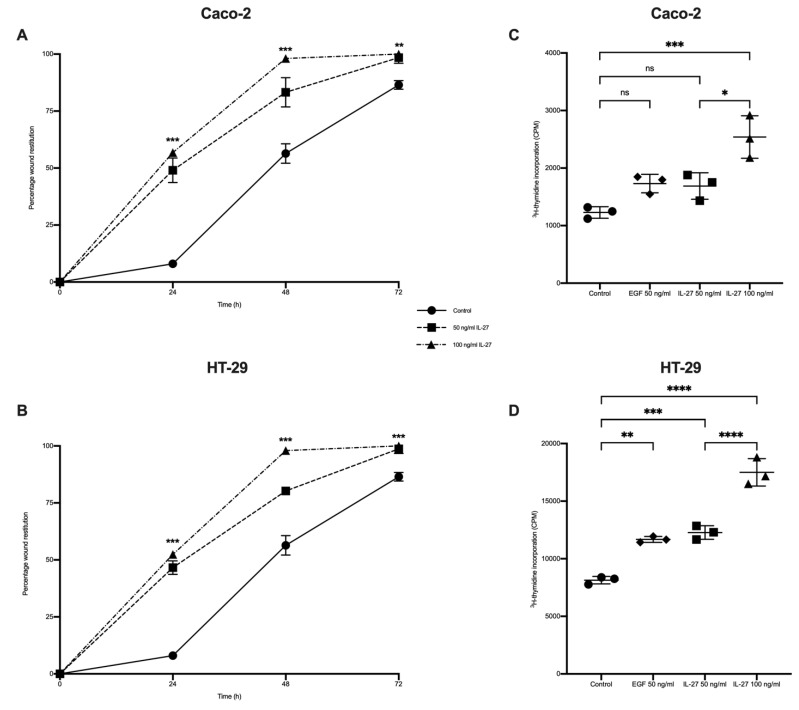
IL-27 effect on gastrointestinal epithelial wound restitution and proliferation. Colonicepithelial cell lines Caco-2 and HT-29 stimulated with 50 and 100 ng/mL IL-27 and growth dynamics were determined in comparison with unstimulated controls by (**A**,**B**) scratch wound assay and (**C**,**D**) ^3^H-thymidine incorporation assessing cell proliferation. Epithelial growth factor (EGF) was positive control. *n* = 3 experimental replicates, each performed in triplicate; data presented as mean ± SD. Statistical analysis with Kruskal–Wallis test (**A**,**B**) and one-way ANOVA with Bonferroni test (**C**,**D**). * *p* < 0.05, ** *p* < 0.01, *** *p* < 0.001 and **** *p* < 0.0001; ns = not significant.

We next assessed wound healing responses in human colorectal cell line culture models. Colorectal cell lines expressed the receptor for IL-27 (IL-27Rα), indicating the capacity to respond to IL-27 stimulation (Figure S1A). Exposure to IL-27 led to enhanced and accelerated wound restitution in both Caco-2 cells and HT-29 cells in a dose-dependent manner (Figure 5A,B, *p* < 0.001). 

Finally, IL-27 influences on gastrointestinal epithelial cell proliferation (Figure 5C,D) were explored. Here, we demonstrate increased ^3^H-thymidine incorporation with rhIL-27 stimulation in a dose-dependent manner with the greatest increase with 100 ng/mL rhIL-27 (*p* < 0.001).

### 3.5. Expression of the Receptor for IL-27 Increases with TNF Exposure

**Figure 6:**
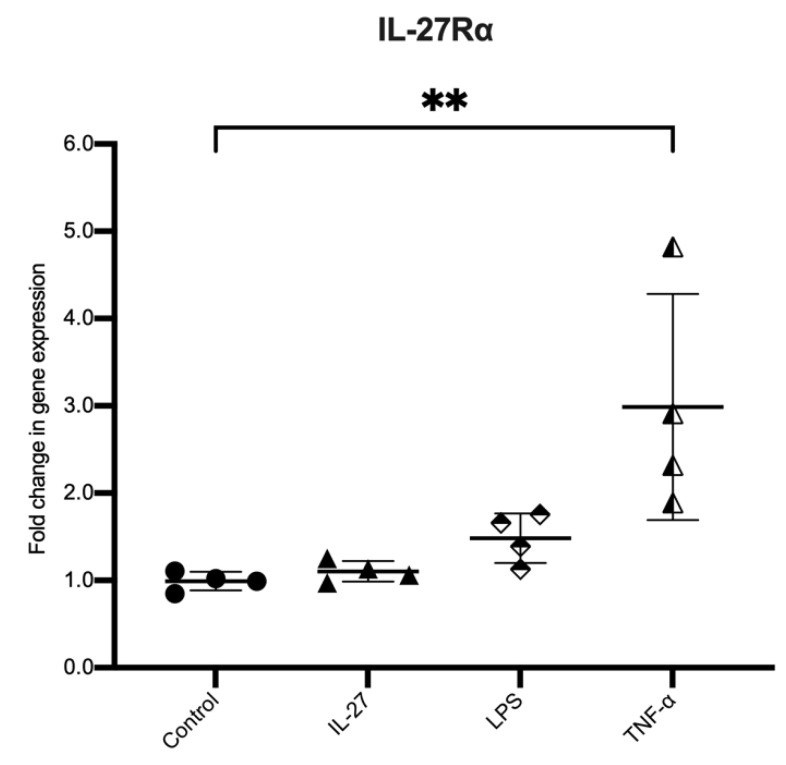
Expression of IL-27Rα in human colonic organoid in a model of IBD. Expression of IL-27Rα in human colonic organoids stimulated with rhIL-27 (100 ng/mL), LPS (500 ng/mL) and TNF-α (100 ng/mL) compared to unstimulated controls. Gene expression normalised to GAPDH; *n* = 3 individual patient-derived organoids stimulated in triplicate for each stimulation, presented as mean ± SD. Statistical analysis with Kruskal–Wallis test. * *p* < 0.05, ** *p* < 0.01, *** *p* < 0.001 and **** *p* < 0.0001; ns = not significant.

Many cytokines modulate cell activity by changing the expression of their own receptor [21]. We assessed whether IL-27 or any other stimulants used in the inflammatory model influenced the expression of IL-27Rα in colonic epithelial organoids (Figure 6). Challenge with IL-27 or LPS did not significantly change IL-27Rα expression. However, exposure to TNF led to a significant increase in expression (*p* = 0.0175). 

## 4. Discussion

Orally delivered IL-27 attenuates IBD in pre-clinical models of the disease [6,7], with indications that the mechanism, in part, could be a direct effect on the gastrointestinal epithelial barrier [7]. A new treatment with concurrent immunoregulatory and direct mucosal healing effects is desirable and in line with current IBD treatment aims of achieving mucosal healing. Patients with IBD who achieve mucosal healing have a lower rate of disease flare, a lower need for hospitalisation and lower rates of colectomy [22,23,24].

Here, we aimed to characterise the effect of IL-27 on the human gastrointestinal epithelial barrier in a human tissue-derived organoid model. This has not been reported previously. We demonstrate that IL-27 regulates expression of key tight junction mediators controlling permeability, leading to restoration of permeability regulation following inflammatory cytokine insult. IL-27 also evokes differential gene expression of mediators to bolster innate immune responses. In addition, we demonstrate that IL-27 induces epithelial barrier restitution following injury. 

These data reveal a new mechanism in human IL-27 biology and has uncovered, using a physiological human tissue-derived organoid model, a previously unrecognised role for IL-27 in the gut. There has been a review of IL-27 immunosuppression in the gut [25]. However, there have been no investigations of IL-27 directly acting on human epithelial cells using organoid culture. Diegelmann and colleagues assessed epithelial responses to IL-27 in DLD-1 colorectal cell line, reporting IL-27-mediated proliferation with increased wound healing, leading to differential gene expression responses across an array of biological pathways [26]. In the skin, another host–environment interface, local CD301b+ cell expression of IL-27 is increased at sites of injury and acts to promote wound repair via increased proliferation of keratinocytes [27]. Zu and colleagues demonstrated increased secretion of the antimicrobial mediator LL-37 from human colonic epithelial cells by IL-27 [28].

Traditionally, investigating human gastrointestinal epithelial barrier biology was challenging due to difficulty with primary cell culture and 2D immortalised cell line models. Although useful for certain investigations, cell line models do not always reflect in vivo function and can biologically change over time. The advent of organoid culture has revolutionised this field [29], offering advantages of near physiological modelling, reflection of patient heterogeneity and a mirror of molecular and morphological identity to source tissue [30,31,32]. There is emerging evidence for the utility of organoid methodology to gain novel insights into the pathogenesis of IBD [33,34,35,36] and for advocating the use of organoid technology to aid assessment of new IBD treatment targets [37,38]. 

We have now applied organoid technology to uncover novel insights into IL-27 cytokine biology. We did not use tissue from individuals with IBD, as it is not clear if the aberrant function of the epithelial barrier in vivo would revert towards a normal phenotype in the in vitro organoid model when released from the toxic inflammatory microenvironment in vivo and provided with a multi-growth factor rich media reflecting homeostatic conditions [38]. Evidence suggests that some characteristics in organoids derived from IBD tissue are retained, including genetic and epigenetic changes [33,39,40,41]. As this has not been fully elucidated and to avoid this confounding, we applied an in vitro inflammatory stimulus to recreate the inflammatory and microbial insults present in IBD, and this approach has been used previously [34,37,42].

Characterising regulatory mechanisms of gastrointestinal epithelial barrier function is important to increase understanding of IBD pathogenesis and to identify new treatment targets. The epithelial barrier has a key role in the pathogenesis of IBD [43]. GWAS studies identified genetic variation in epithelial-associated genes as a risk signature in IBD [44,45]. There is evidence that a ‘leaky’, more permeable epithelial barrier is present in active inflammation. IBD is associated with thinner mucus layer, reduced MUC2 secretion and Paneth cell dysfunction [38]. The initial trigger injury in IBD remains elusive. It is not clear if the changes in epithelial barrier structure and function are cause or effect from the mucosal inflammation or microbial dysbiosis. Altered permeability can predict risk of relapse, suggesting that this is an early step in disease pathogenesis [46,47]. Individuals with Crohn’s disease and their first-degree relatives with no IBD had altered permeability, although the consequence of this is unclear [48]. Patients with asymptomatic Crohn’s disease demonstrate increased intestinal permeability that precede onset of clinical symptoms [49]. Here, we show that IL-27 supports gastrointestinal barrier regulation of permeability and homeostasis in the face of an inflammatory insult via multi-factorial mechanisms. Further work to establish tight junction protein localisation by microscopy and exploration of the cell signalling cascades that lead to these effects would increase understanding further. Expanding the repertoire of tight junction proteins studied would also offer further biological insights. For example, the claudins are a large family of proteins with 27 members, involved in permeability, barrier integrity and proliferation [50], with several members implicated in IBD pathogenesis, such as the pore sealing claudin-3. It is likely that IL-27 impacts on the expression and function of additional mediators within the gastrointestinal microenvironment that could impact on epithelial barrier structure and function. An example of this is indoleamine 2,3-dioxygenase (IDO). This enzyme, which metabolises one mechanism of tryptophan metabolism, is important in gut homeostasis with impacts on bacterial function and immunoregulatory actions across a wide array of immune cell subsets [51]. IDO has been implicated in IL-27 responses in cancer cells [52]. In addition, IDO is expressed by colonic epithelial cells and increased in IBD [53]. This increased expression was identified around sites of ulceration, raising the possibility IDO could be implicated in epithelial repair. The full impact on the epithelial barrier is not known. 

With regard to translational potential, the IL-27 effects on the epithelial barrier could be exploited for a novel therapeutic strategy for IBD. We show that the expression of the IL-27Rα receptor subunit is increased in colonic epithelial cells under inflammatory conditions, suggesting the need for an inflammatory microenvironment to maximise the effect of immunoregulatory and barrier protective functions. In line with this, we previously found that pre-treatment of mice with orally delivered IL-27 prior to onset of TNBS mediated colitis did not alter disease induction. However, IL-27 was highly effective as a treatment once inflammation was established [7]. This may indicate that IL-27 would be particularly effective in disease flare and may reduce the need for use of steroid therapy or be exploited as a colon rescue therapy to avoid colectomy in acute severe colitis. 

IBD is associated with an increased risk of colon cancer, dependant on duration and extent of the inflammation [54]. The finding here that IL-27 increases epithelial cell proliferation raises the question of whether this may impact on the development of malignancy. A single-nucleotide polymorphism (rs153109) in the IL-27 gene is associated with risk of colorectal cancer with carriage of the G allele conferring risk [55]; however, the biological consequences of this allele on IL-27 expression and function is unclear. IL-27 can exert an anti- or pro-tumorigenic role, dependant on site, stage and other microenvironmental influences [56]. The anti-malignant effects appear predominant across multiple different malignancies and are multi-factorial, including anti-angiogenic effects, reduced migration and evasion and effects on immune responses [56]. This has led to the investigation of IL-27 as a therapeutic for prostate cancer [57]. In the colon, Cui and colleagues [58] demonstrated a protective role for IL-27 in a colitis-associated colorectal cancer murine model using an IL-27 Rα knockout model. It is not known if IL-27 or its specific receptor IL-27Rα is increased in human colorectal cancer, nor the biological consequence of this. Here, we show that IL-27 increases expression of the essential epithelial protein E-cadherin, a core constituent of adheren junctions to maintain barrier function and control cell–cell adhesion. E-cadherin is a tumour suppressor through influences of Wnt signalling [59]. The influences of IL-27 and mechanisms of action on IBD-associated colorectal carcinogenesis will need further exploration in human colorectal cancer models. 

## 5. Conclusions

In conclusion, this study characterised the novel biology of IL-27 in human colonic epithelial cells and translated previous data from pre-clinical models of IBD to human tissue modelling. The findings from this study provide proof of concept of the utility in further developing IL-27 as a new therapy for IBD. 

## Figures and Tables

**Table 1:** Epithelial junctional protein expression data in human colonic organoids in an inflammatory micro-environment.

	Relative Protein Expression			
	Claudin-2	Claudin-4	Occludin	E-Cadherin
IL-27	0.835 (±0.097)	1.180 (±0.024)	1.233 (±0.108)	1.148 (±0.121)
LPS + TNF	1.037 (±0.068)	0.737 (±0.070)	0.939 (±0.017)	0.737 (±0.070)
IL-27 + LPS + TNF	0.774 (±0.097)	1.182 (±0.020)	1.095 (±0.075)	1.152 (±0.070)

Data are presented as mean change in relative protein expression compared to unstimulated controls, normalised to GAPDH ± SD, *n* = 3.

## Data Availability

All data related to this study are contained within the manuscript. Data can be obtained from the authors on request.

## Supplementary Materials

The following supporting information can be downloaded at: https://www.mdpi.com/2079-7737/11/3/427/s1, Figure S1: Expression of IL-27Rα and TLR4 in human colonic organoid in a model of IBD.
