# Supplementary material for: Interleukin-27 Regulates the Function of the Gastrointestinal Epithelial Barrier in a Human Tissue-Derived Organoid Model
Source: Biology (Basel). 2022 Mar 11;11(3):427. doi: 10.3390/biology11030427 (PMC8945023; doi:10.3390/biology11030427)
Supplement: Supplementary file 1 [file biology-11-00427-s001.zip › biology-1589659-supplementary.pdf]

Supplementary Figure S1 – **Expression of IL-27Rα and TLR4 in human colonic organoid in a model of IBD**

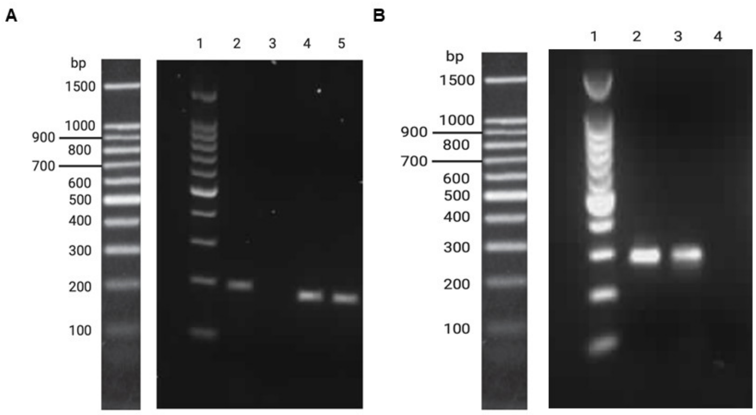

(**A**) Expression of IL-27Rα confirmed by PCR amplification of RNA isolated from human patient derived organoids. DNA size ladder (lane 1), positive control (Caco-2 cDNA) (lane 2), negative control (lane 3), human intestinal epithelial organoids (lanes 4-5).(**B**) Expression of TLR4, DNA size ladder (lane 1), positive control (Caco-2 cDNA) (lane 2), human intestinal epithelial organoids (lanes 3), negative control (lane 4).
